# The Austrian MS database and the Austrian MS cohort: A national effort towards data harmonization and prospective data collection

**DOI:** 10.1007/s00508-025-02689-2

**Published:** 2026-01-12

**Authors:** Gabriel Bsteh, Fabian Föttinger, Markus Ponleitner, Klaus Berek, Franziska Di Pauli, Bettina Heschl, Sebastian Wurth, Florian Deisenhammer, Christian Enzinger, Thomas Berger, Michael Khalil, Harald Hegen

**Affiliations:** 1 Department of Neurology, Medical University of Vienna, Vienna, Austria; 2 Comprehensive Center for Clinical Neurosciences & Mental Health, Medical University of Vienna, Vienna, Austria; 3 Department of Neurology, Medical University of Innsbruck, Innsbruck, Austria; 4 Department of Neurology, Medical University of Graz, Graz, Austria

**Keywords:** Multiple sclerosis, Infrastructure, Common data element, Data collection, Data processing

## Abstract

**Background:**

Multiple sclerosis (MS) is treated with various disease-modifying therapies (DMTs) with differing efficacy and risks, yet the disease course varies markedly within and between individuals. Defining optimal treatment strategies through randomized trials is impractical because of the required sample sizes, costs and long follow-up. Large multicenter registries and prospective observational cohorts are therefore essential but demand harmonized, standardized, user-friendly data capture with rigorous quality control and data protection.

**Objective:**

This project aims to establish standardized, nationwide MS data collection in Austria.

**Methods:**

The project consists of five key components: (i) harmonization of data collection, (ii) creation of infrastructure for data sharing, (iii) retrospective harmonized data collection (Austrian MS Database, AMSD), (iv) prospective harmonized data collection (Austrian MS Cohort, AMSC) and (v) aggregated analyses.

**Results:**

A comprehensive set of harmonized common data elements (CDE) comprising clinical and paraclinical data was developed and a common data collection infrastructure was generated using the web-based Research, Documentation, and Analysis platform (webRDA), an innovative data capture, processing and analysis system provided by the Medical University of Vienna offering pseudonymized storage of data supported by a robust permissions system fulfilling legal data protection and ethical requirements.

The AMSC is set up as a standardized prospective collection of demographic, clinical, epidemiological, psychosocioeconomic, magnetic resonance imaging (MRI), and optical coherence tomography (OCT) data as well as body fluids.

**Conclusion:**

The AMSD and AMSC will facilitate the evidence-based development of prognostic biomarkers, individualized therapy strategies and treatment sequences based on a high-quality, population-based dataset of more than 8000 people with MS.

**Supplementary Information:**

The online version of this article (10.1007/s00508-025-02689-2) contains supplementary material, which is available to authorized users.

## Background

Multiple sclerosis (MS) is an immune-mediated, chronic inflammatory, demyelinating disease of the central nervous system and is the leading cause of neurological disability in young adults [1]. Over the past three decades, an ever-growing armamentarium of disease-modifying therapies (DMTs) has emerged, enabling effective suppression of disease-activity [2].

These DMTs vary considerably in terms of efficacy and associated risks. With the availability of high-efficacy DMTs, the goal of MS treatment has shifted from simply managing the disease course (reducing relapses, disability progression and radiological signs of disease activity) to achieving suppression of disease activity below detectable levels [3]; however, it is essential to recognize that MS exhibits extremely variable intraindividual and interindividual courses. Some people with MS (pwMS) experience highly active disease with breakthrough activity even under high-efficacy DMTs (HE-DMT), while others have a mild disease course that may not necessitate a potentially risky, psychologically burdensome and costly DMT [4]. Conversely, a growing perspective advocates HE-DMT in the majority or even all pwMS. The increasing array of therapeutic options also raises questions about the optimal sequencing of these treatments.

Currently, there is a lack of direct prospective comparative studies between individual treatments, treatment sequences and treatment strategies to address these questions in an evidence-based manner. A significant challenge is that the clinical trials for DMTs, conducted under strict inclusion criteria, exclude a relevant proportion of pwMS [5]. Furthermore, the impact of DMTs on various aspects of MS pathology and the influence of factors such as lifestyle, psychosocioeconomic background, comorbidities and aging are not addressed in these studies.

Due to the enormous sample sizes and associated costs, conducting such studies with the classical randomized controlled trial design is impractical. Thus, the only feasible approaches for generating meaningful insights into this area lie in large-scale, multicenter registries and well-characterized prospective long-term observational cohorts combining the systematic acquisition of clinical data, imaging and laboratory measures; however, for such projects to yield valid results, two essential prerequisites must be met: a harmonized, universally accepted approach for data collection, wherein all contributors collect and document data in a consistent manner and a data infrastructure that enables standardized, comprehensive and user-friendly data collection while adhering to data protection and security requirements and enabling quality control. Finally, such registries and databases must be designed to accommodate contributions from as many eligible centers as possible to maximize sample size and population diversity and the collected data should be accessible for analysis by a broad research community (data sharing). In the field of MS, there have been several such projects such as various national MS registries and the well-known MS Base registry [6]; however, these projects cover a highly limited set of common variables, e.g., basic demographic data, clinical course with relapses and expanded disability status scale (EDSS) as well as the type, timing and duration of DMTs, only sometimes including even basic paraclinical data such as the number and dynamics of MRI lesions. Furthermore, these datasets were not harmonized, meaning they were collected and documented in different ways and are subject to only rudimentary quality control, resulting in compromised data quality and limited data sharing capability.

In Austria, the nationwide network of certified MS centers, established by the Austrian Society of Neurology (ÖGN), provides high-quality care and documentation; however, the currently established Austrian MS Therapy Registry (AMSTR) only includes pwMS receiving a DMT (excluding interferon beta or glatiramer acetate formulations), leaving a significant portion of the more than 14,000 pwMS in Austria unrecorded [7].

In 2016 the Medical Universities of Vienna, Innsbruck and Graz initiated a multicenter biomarker project in MS with four specific aims: (i) to establish and maintain a long-term cohort of pwMS in Austria, (ii) to systematically follow these pwMS with standardized collection of demographic, clinical, MRI and body fluid data and (iii) to uphold and enhance the high standard of MS care in Austria by evaluating the long-term efficacy and safety profiles of available DMT.

Based on these previous efforts and collaborative structures, this research project aims (i) to establish a harmonized, nationwide collection of a broad array of real-world data on pwMS in Austria (Austrian MS Database, AMSD) and (ii) to evolve the prospective long-term observational comprehensive cohort study of demographic, clinical, epidemiological, psychosocioeconomic, MRI and OCT data as well as body fluids (Austrian MS Cohort, AMSC), in order to facilitate nested projects on prognostic indicators, biomarkers, individualized treatment strategies and treatment sequences.

## Methods

The project consists of five key components:

1. Harmonization of data collection.
2. Infrastructure creation for collection, management and sharing of data.
3. Retrospective and prospective harmonized collection of real-world data (AMSD).
4. Prospective harmonized collection of comprehensive high-quality data (AMSC).
5. Analyses of aggregated data.

### Harmonization of data collection

The harmonization of data collection was coordinated by the AMSD core centers comprised of the Departments of Neurology of the Medical Universities of Vienna, Innsbruck and Graz.

**Fig. 1:**
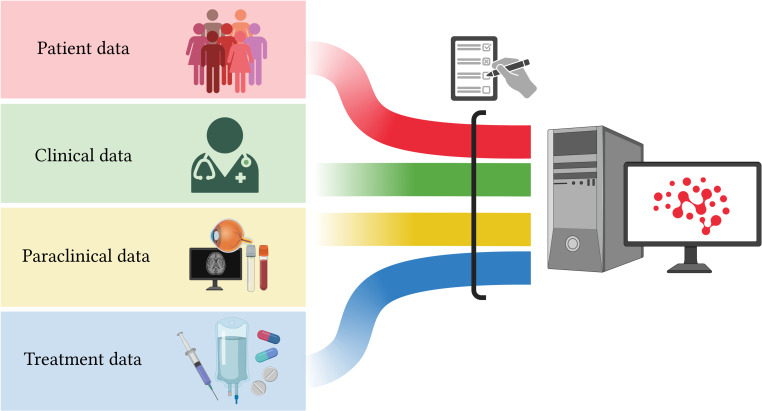
Overview of the components of the Austrian Multiple Sclerosis Database

The core centers developed a draft of common data elements (CDE), which were then discussed with representatives from MS clinics of all participating sites and refined over multiple iterations into a final set of CDEs including precise definitions of documentation and coding of data. This final dataset serves as the harmonized foundation for data collection within the AMSD and encompasses patient data (demographics, family history, education, occupation, social environment, consumption of alcohol and nicotine), clinical data (MS disease course, initial symptoms, relapses, relapse remission, expanded disability status scale, EDSS, comorbidities, concomitant medications, pregnancies, vaccination status), paraclinical data (cerebral/spinal magnetic resonance imaging, MRI, optical coherence tomography, OCT, serum and cerebrospinal fluid parameters/biomarkers) and treatment (relapse treatment, DMT including side effects, symptomatic treatment) (Fig. 1). A complete summary of AMSD-CDE is provided in the supplements (Supplemental File 1).

**Fig. 2:**
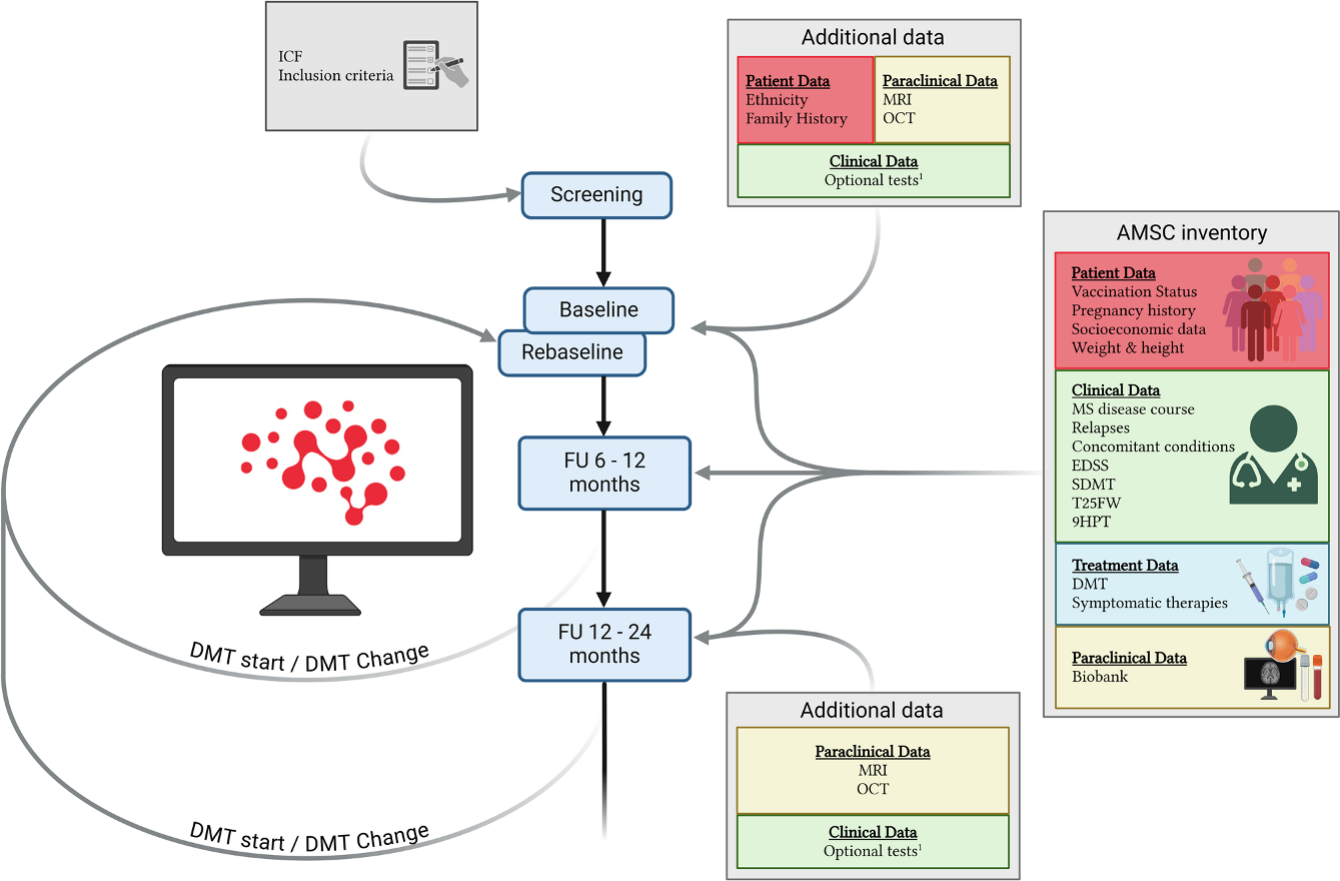
Overview Austrian Multiple Sclerosis Cohort. *AMSC* Austrian Multiple Sclerosis Cohort, *DMT* disease-modifying treatment, *EDSS* expanded disability status scale, *FU* follow-up, *ICF* informed consent form, *MRI* magnetic resonance imaging, *OCT* optical coherence tomography, *SDMT* symbol digit modalities test, *T25FW* timed 25-foot walk test, *9HPT* 9-hole peg test. ^1^Optional tests include: *BDI* Beck Depression Inventory, *FSMC* Fatigue Scale for Motor and Cognitive Fatigue, *HADS* Hospital Depression and Anxiety Scale, *MSIS-29* Multiple Sclerosis Impact Scale, *PeRiCoMS* personality, risk perception and coping in MS battery,* SCOPE-MS AT* AMSC socioeconomic inventory

### Infrastructure for data sharing

The data infrastructure is developed as a web-based Research, Documentation and Analysis (RDA) database, a non-commercial platform supported by the Medical University of Vienna enabling collection of research-relevant data in specifically designed modular registries [8]. This data pool is continuously and automatically supplied with specifically selected documented routine data, which are fed into RDA registries. The combined use of both data sources ensures enhanced scientific utilization and includes functionalities, such as form-based documentation, data collections for multicenter registries, patient-specific and pseudonymized documentation and the automated transfer of data from various sources. From a data protection perspective it is particularly noteworthy that the RDA offers pseudonymized web-based documentation for multicenter registries, supported by a robust permissions system (including read, edit and query rights, along with the logging of all access events).

The AMSD-RDA comprises all agreed AMSD-CDE, which creates a virtual database integrating data from all participating centers into a unified data model.

Each core center as well as each future participating center have reading and writing access only to the patient data entered by its own center. Centers not able to use the web-based RDA due to local legal or technical restrictions can also use an off-line version of the AMSD using the same CDE to collect data. Each participating MS center signs a data-sharing agreement that ensures data protection and grants end users reading and writing access exclusively to the patient data entered by the respective center.

### Retrospective harmonized data collection and data management (AMSD)

At all participating MS centers, the AMSD is populated retrospectively using pre-existing local databases and patient records, as well as prospectively following inclusion into the AMSD. The dataset includes all patients who meet the following criteria:

1. Diagnosis of multiple sclerosis according to the McDonald criteria, in the version applicable at the time of initial diagnosis (2001/2005/2010/2017/2024) [9–13].
2. Initial diagnosis on or after 1 January 2001.

As of November 2025, 5800 patients have already been included into the AMSD.

Data collection is conducted in a pseudonymized form, with each patient assigned a unique, center-specific sequential ID number. The datasets can only be linked to identifiable patient information through a subject log, which remains exclusively at each center.

Retrospective data collection is performed using the existing database from the core centers by trained academic personnel and who are supervised by the AMSD project core team to ensure standardized and high-quality data collection [14]. Quality assurance is provided through automated plausibility and completeness checks performed at the point of data entry. Cross-site monitoring is done by double data extraction on a 5% sample of entries and a plausibility check by the AMSD project core team on another 5% sample. Additional quality assurance measures comprise periodic harmonization workshops, review of variable distributions across centers and automated detection of outliers and inconsistencies. Planned analyses will follow standardized procedures for handling missing data to ensure robustness and comparability across sites. Moreover, cross-validation studies with other registries such as the Swiss MS cohort are planned.

The AMSD was approved by the ethics committee of the coordinating center at the Medical University of Vienna (ethical approval number: 1668/2023) as well as the ethics committees of the Medical University of Innsbruck (approval number: 1050/2023) and Graz (approval number: 31-432 ex 18/19). As data are extracted from pre-existing local databases and patient records obtained in routine practice, the need for written informed consent from study participants was waived by the ethics committees.

### Prospective harmonized data collection (Austrian MS cohort, AMSC)

The Austrian MS Cohort (AMSC) is an evolution of the previous research projects initiated between the core centers in Vienna, Innsbruck and Graz, which began recruitment in 2016, with 4 specific aims: (i) to establish and maintain a long-term cohort of pwMS in Austria, (ii) to systematically follow these pwMS with standardized collection of demographic, clinical, epidemiological, psychosocioeconomic, MRI and OCT data as well as body fluids and (iii) to uphold and enhance the high standard of MS patient care in Austria by evaluating the long-term efficacy and safety profiles of available DMT for MS (Table 1, Fig. 2). As of November 2025, 580 patients have already been included into the AMSC.

To be included in the AMSC, individuals must be diagnosed with either relapsing-remitting MS (RRMS), secondary progressive MS (SPMS), or primary progressive MS (PPMS) according to the 2024 revised McDonald criteria [13]. Patients diagnosed with clinically isolated syndrome (CIS), radiologically isolated syndrome (RIS), neuromyelitis optica spectrum disorder (NMOSD) and myelin oligodendrocyte glycoprotein antibody-associated disease (MOGAD) may also be included [15–17].

Patients are enrolled in the AMSC only after signing written informed consent. Every effort is made to minimize dropouts. If a patient relocates within Austria, the original center coordinates a consultation with the nearest participating center.

Data collected within the AMSC are entered and managed within the RDA framework. Data quality is subject to several automatic and manual internal quality checks including annual quality control checks of a random sample of 5% records and another random sample of 10% newly added records. When inconsistencies are observed, queries are sent to the respective center until the discrepancy is successfully solved. To minimize the risk of duplicate entries, e.g. when patients transfer between centers, we have implemented an automated flagging procedure based on records with the same sex, year of birth and year of diagnosis, prompting a review for possible duplication. If the center confirms that the patient is already present in the registry, the existing record can be reassigned to the new center, allowing the receiving site to continue documentation without creating a duplicate entry.

The AMSC was approved by the ethics committee of the coordinating center at the Medical University of Vienna (approval numbers: 2195/2016 and 1668/2023), as well as the ethics committee of the Medical University of Innsbruck (approval number: 1050/2023) and Graz (approval number: 31-432 ex 18/19) and will be approved as well by independent ethics committees at each participating center.

#### Collection of demographic, epidemiological, clinical and psychosocioeconomic data

At baseline variables collected include sex, date of birth, height, weight, ethnicity, family history, pregnancy history, consumption of alcohol and nicotine, date and type of first MS symptoms, date and number of relapses, type of relapses, relapse treatment and remission, date of diagnosis, MS disease course, date of progression onset (if applicable), current and prior DMT as well as concomitant medical conditions, medications and vaccination status. Standardized clinical assessments, including EDSS calculation with functional system scores, the timed 25-foot walk test (T25FW), 9 hole peg test (9HPT) and symbol digit modalities test (SDMT) are conducted by certified raters [18–20].

In addition, while not mandatory within the AMSC, we aim to obtain the following clinical scales and questionnaires at baseline: Beck Depression Inventory (BDI) and/or the Hospital Anxiety and Depression Scale (HADS) to quantify depression and/or anxiety, Fatigue Scale for Motor and Cognitive Functions (FSMC) to quantify fatigue, Multiple Sclerosis Impact Scale (MSIS-29) to quantify MS-related quality of life, the PeRiCoMS test battery to quantify personality, risk awareness, and coping strategies, and the AMSC socioeconomic inventory (SCOPE-MS AT) to retrieve data on socioeconomic and occupational status, housing and living conditions, healthcare access and medical expenses, support network and social life, health literacy and self-management, psychosocial and emotional well-being [21–23].

Each patient is followed up every 6 or 12 months ± 45 days as determined by the treating physician. At each visit, data on the occurrence of relapses, disability worsening (as measured by EDSS, T25FW, 9HPT, SDMT), initiation or interruption of DMT, DMT-related adverse events, weight, additional medical conditions and concomitant medications are recorded. In addition, while again not mandatory within the AMSC, we aim to obtain the BDI and/or HADS, FSMC, MSIS-29 at each follow-up visit and within 3–6 months after each DMT start (rebaseline). The PeRiCoMS and SCOPE-MS AT are aimed to be performed biennially and at rebaseline.

#### Magnetic resonance imaging

Acquisition of MRI based on a fixed schedule and protocol is not mandatory within the AMSC; however, our goal is to obtain cranial MRI annually or biennially from as many patients as possible (ideally all). In addition, we aim to conduct cranial MRI within 3–6 months after DMT initiation or change (rebaseline). All scans should be performed within ±28 days of clinical data and sample collection in agreement with MAGNIMS-CMSC-NAIMS (Magnetic Resonance Imaging in MS – Consortium of MS Centers – North American Imaging in MS) consensus recommendations [24]. A state-of-the-art MRI protocol, already established at the core centers Vienna, Innsbruck and Graz in 2016 and to be agreed upon by all contributing AMSC centers, includes high-resolution isotropic T1 (3D) without gadolinium (Gd) and postintravenous Gd contrast, 3D-FLAIR (fluid attuated inversion recovery) isotropic (or 2D if 3D is not feasible) and susceptibility-weighted imaging (SWI) (for the detailed protocol see Supplemental File 2). We aim to perform MRI scans primarily at 3 T field strengths, but 1.5 T scanners are accepted as well. Advanced MRI sequences (e.g., diffusion tensor imaging (DTI), magnetization transfer ratio (MTR), etc.) may be included as part of nested projects. The MRI readings are performed and stored locally at the AMSC core centers and can be uploaded securely for quality control, centralized storage, back-up and standardized analyses within the Neurodesk platform [25].

#### Optical coherence tomography

The use of OCT is not mandatory within the AMSC; however, our goal is to perform spectral-domain OCT in accordance with the Austrian Network for OCT in MS (AN-OCT-MS) consensus for each AMSC participant at diagnosis (if applicable), before each DMT start, within 3–6 months after each DMT start (rebaseline) and then annually to biennially [26]. Routine parameters obtained include peripapillary retinal nerve fiber layer (pRNFL) and ganglion cell and macular inner plexiform layer (GCIPL) thicknesses separate for each eye with documentation of optic neuritis history. All examinations are checked for sufficient quality using OSCAR-IB criteria. Scans from patients/eyes with diagnoses of ophthalmological (e.g., myopia greater than −6 diopters, optic disc drusen, glaucoma), neurological or drug-related causes of retinal damage not attributable to MS are excluded [26, 27]. The OCT readings are performed and stored locally at the AMSC core centers and can be uploaded securely for quality control, centralized storage, back-up and standardized analyses with the Vienna Reading Center (VRC) serving as central reference [28].

#### Laboratory parameters and biobanking

At each AMSC visit, serum and plasma samples are collected within the clinical routine (as detailed in Supplemental File 3) and for biobanking according to standardized guidelines. For the purpose of genotyping, DNA-EDTA whole blood samples are collected at least once from each AMSC participant. From lumbar punctures performed as part of routine clinical care, routine cerebrospinal fluid (CSF) parameters are documented and CSF samples are also collected for biobanking. Serum, plasma, DNA-EDTA whole blood, and CSF samples are then immediately stored at −80 °C in biobanks established at the core centers (Vienna: approval number 2195/2016, Innsbruck: approval number 1050/2023, Graz: approval number 31-432 ex 18/19) in accordance with international consensus guidelines [29, 30]. A fully pseudonymized linkage process is used to connect decentralized biobank samples with registry data. Control groups will be defined based on specific study questions but following standardized guidelines [31].

### Governance and aggregated data queries

**Fig. 3:**
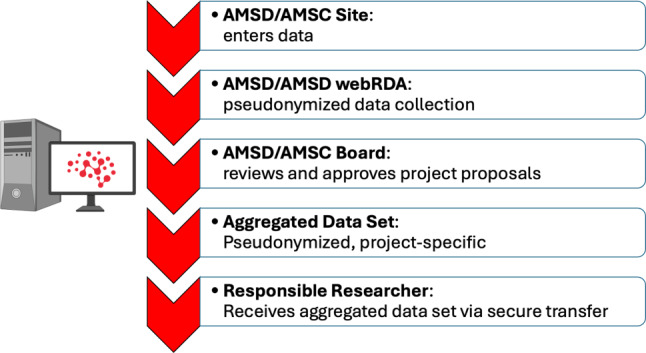
Data governance Austrian Multiple Sclerosis Database and Cohort. *AMSC* Austrian Multiple Sclerosis Cohort, *AMSD* Austrian Multiple Sclerosis Database

The AMSD/AMSC Board, which consists of a fixed representative from each of the core centers in Vienna, Innsbruck, and Graz, along with two rotating independent representatives from contributing centers (only one from each center allowed), oversees all aspects of conducting scientific nested research projects within the AMSD/AMSC (Fig. 3).

Each project proposal must be submitted by the responsible project leader in writing to the AMSD/AMSC Board via a standardized application form accompanied by a brief protocol (including research question, inclusion and exclusion criteria, required data, and analysis plan) to contact@amsc.at. Each request is reviewed based on predefined evaluation criteria by the AMSD/AMSC Board. Projects require unanimous approval from the AMSD/AMSC Board with mandatory documentation of all decisions to ensure transparent, standardized decision-making for data use.

The webRDA software enables cross-center aggregated data queries and analyses within the entire AMSD/AMSC. Centers not able to use the webRDA due to local legal or technical restrictions can obtain data based on identical local queries. Each project proposal from within the AMSD/AMSC network involving an aggregated AMSD/AMSC data query must be submitted to the Ethics Committee and the data-clearing committee of the Medical University of Vienna for review and approval. International data and sample sharing is also possible and explicitly encouraged by the AMSD/AMSC. For projects outside the AMSD/AMSC network including non-EU countries, each collaboration additionally requires a project-specific data transfer agreement (DTA).

To ensure scientific quality as well as ethical and data protection integrity, aggregated database queries can only be conducted by the coordinating center at the Medical University of Vienna.

Upon approval by both the AMSD/AMSC Board and the Ethics Committee, the requested data are provided in pseudonymized form via secure data transfer (safe cloud or personal collection of a storage device) to the respective project leader.

Project leaders are responsible for the ongoing security and integrity of the data, with any sharing or use outside the approved project strictly prohibited. Project leaders are also required to (i) provide semi-annual progress reports and a final report on the analysis results, (ii) submit any planned analyses based on AMSD/AMSC data, either partially or entirely, to the AMSD/AMSC Board for review and approval prior to publication, (iii) explicitly and visibly acknowledge the AMSD/AMSC as the data source in all publications derived partially or entirely from AMSD/AMSC data and (iv) recognize contributing MS centers in line with good scientific practice guidelines, either through acknowledgments or co-authorships.

## Perspectives

This project establishes the foundation for standardizing collection of comprehensive data of pwMS across Austria providing a data protection-compliant system for gathering population-based data in accordance with FAIR data principles (findable through standardized metadata, accessible via governed request procedures, interoperable through structured data formats and reusable under clearly defined conditions).

Through a coordinated, consensus-driven approach, it substantially enhances the interconnectedness of MS centers throughout Austria.

Due to the interindividual and intraindividual heterogeneity in MS courses together with the ever-growing complexity of biomarker and treatment options, establishing an infrastructure for comprehensive harmonized collection of population-based data of pwMS is the most promising approach for facilitating evidence-based development of individualized treatment strategies. In comparison with major international MS registries (e.g., MSBase, MS PATHS, NARCOMS, UK MS Register, Swiss MS Cohort), the AMSD/AMSC captures a largely overlapping set of core clinical variables, enabling future interoperability. At the same time, our initiative extends beyond these CDE by systematically integrating more detailed clinical characterization, OCT imaging, biobanking and detailed socioeconomic information. This broader data structure enhances both translational research capacity and population-level analyses, setting the AMSD/AMSC apart within the international registry landscape, while simultaneously enabling internation collaboration and integration into the European Health Data Space.

This project opens a myriad of scientific opportunities to analyze a plethora of diverse questions related to the diagnosis, treatment and prognosis of pwMS, based on a high-quality data foundation. Planned analyses include studies extending established concepts of treatment response, such as no evidence of disease activity (NEDA), through incorporation of OCT metrics and serum/plasma biomarkers. Additional research priorities involve validation of prognostic biomarkers (e.g., age as a determinant of high-efficacy DMT choice, OCT and fluid biomarkers), and development of multimodal prediction models using artificial intelligence (AI)-based approaches. Furthermore, we plan to evaluate socioeconomic factors as predictors of disease progression and treatment response.

For patients, benefits arise from both the high level of data security provided by the RDA system (including the webRDA application) and the improved quality assurance resulting from enhanced center connectivity as well as strengthened evidence-based practices, which will further advance the quality of clinical care. Although patient organizations were not involved in the initial design or governance, incorporating structured patient and patient-organization representation is a key objective for future development of the AMSD/AMSC.

Finally, by integrating into the RDA infrastructure, which is inherently scalable across the European Union, this project contributes to the creation of a digital ecosystem as part of a European Health Data Space for MS.

**Table 1:** Austrian Multiple Sclerosis Cohort overview

	Screening	Baseline	Follow-up every 6–12 months	Follow-up every 12–24 months	Rebaseline (3–6 months after DMT start/change)
*Written informed consent*	X	**–**	**–**	**–**	**–**
*Inclusion criteria*	X	**–**	**–**	**–**	**–**
*History*	**–**	X	**–**	**–**	**–**
Ethnicity	**–**	X	**–**	**–**	**–**
Family history	**–**	X	**–**	**–**	**–**
MS disease course	**–**	X	X	X	X
Relapses	**–**	X	X	X	X
DMT	**–**	X	X	X	X
Concomitant medication	**–**	X	X	X	X
Concomitant conditions	**–**	X	X	X	X
Vaccination status	**–**	X	X	X	X
Pregnancy history	**–**	X	X	X	X
*Height*	**–**	X	X	X	X
*Weight*	**–**	X	X	X	X
*EDSS*	**–**	X	X	X	X
*SDMT*	**–**	X	X	X	X
*T25FW*	**–**	X	X	X	X
*9HPT*	**–**	X	X	X	X
*BDI or HADS (optional)*	**–**	X	X	**–**	X
*FSMC (optional)*	**–**	X	X	**–**	X
*MSIS-29 (optional)*	**–**	X	X	**–**	X
*PeRiCoMS (optional)*	**–**	X	**–**	X	X
*AMSC socioeconomic inventory (SCOPE-MS-AT, optional)*	**–**	X	**–**	X	X
*Brain MRI*	**–**	X	**–**	X	X
*Spinal cord MRI (optional)*	**–**	X	**–**	X	X
*OCT*	**–**	X	**–**	X	**–**
*Biobank*	**–**	X	X	X	X

*AMSC* Austrian Multiple Sclerosis Cohort. *BDI* Beck Depression Inventory. *DMT* disease-modifying treatment. *EDSS* expanded disability status scale. *FSMC* Fatigue Scale for Motor and Cognitive Fatigue. *HADS* Hospital Depression and Anxiety Scale. *MRI* magnetic resonance imaging. *MSIS-29* Multiple Sclerosis Impact Scale. *OCT* optical coherence tomography. *PeRiCoMS* personality, risk perception and coping in MS battery. *SDMT* symbol digit modalities test. *T25FW* timed 25-foot walk test. *9HPT* 9-hole peg test

## Supplementary Information

Complete summary of Austrian Multiple Sclerosis Database (AMSD) common data elements (CDE)

Magnetic Resonance Imaging (MRI) acquisition protocol in the Austrian Multiple Sclerosis Cohort (AMSC)

Overview of laboratory parameters collected in the Austrian Multiple Sclerosis Cohort (AMSC)
