# Supplementary material for: The Austrian MS database and the Austrian MS cohort: A national effort towards data harmonization and prospective data collection
Source: Wien Klin Wochenschr. 2026 Jan 12;138(13-14):394–402. doi: 10.1007/s00508-025-02689-2 (PMC13375655; doi:10.1007/s00508-025-02689-2)
Supplement: Supplementary file 2 — Magnetic Resonance Imaging (MRI) acquisition protocol in the Austrian Multiple Sclerosis Cohort (AMSC) [file 508_2025_2689_MOESM2_ESM.pdf]

Table of contents

\\NEURO
HEAD_64
Standard
BIGWIG
AAhead_#beta1
t2_space_dark-fluid_sag_iso
t2_space_sag_iso_SMS2_2rpt_ThuGut_BT#6
3DT1_sag
SWI_MIU
resolve_diff_224
AAspine_scout
t2_tse_sag_HWS
3DT1_tra_KM
T1_space_fs_BB_sag

\\NEURO\HEAD_64\Standard\BIGWIG\AAhead_#beta1

TA: 14 sec Coil Selection: Auto Voxel Size: 1.6×1.6×1.6 mm³ Acc:: 3 Rel. SNR: 1.00

Properties

Start measurement without further preparation	Off
Wait for User to Start	Off
Start measurements	Single Measurement
Prio Recon	Off
Auto Open Inline Display	Off
Auto Close Inline Display	Off
Load Images to MR View&GO	On
Auto Store Images	On
Load Images to Stamp Segments	Off
Load Images to Graphic Segments	On
Graphic segment	Default
Inline Movie	Off

Routine

Slab Group	1
Slabs	1
Distance Factor	20 %
Position	Isocenter
Orientation	Sagittal
Phase Encoding Dir.	A >> P
Slices per Slab	128
Phase Oversampling	0 %
Slice Oversampling	0.0 %
FoV Read	260 mm
FoV Phase	100.0 %
Slice Thickness	1.6 mm
TR	3.2 ms
TE	1.37 ms
Averages	1
Concatenations	1
AutoAlign	Head
Coil Elements	HC1-7

Contrast - Common

TR	3.2 ms
TE	1.37 ms
Flip Angle	8 deg
Fat-Water Contrast	Standard
Contrasts	1
Reconstruction	Magnitude

Contrast - Dynamic

Dynamic Mode	Standard
Measurements	1
Time to Center	6.2 s

Resolution - Common

FoV Read	260 mm
FoV Phase	100.0 %
Slice Thickness	1.6 mm
Base Resolution	160
Phase Resolution	100 %
Slice Resolution	69 %
Trajectory	Cartesian

Resolution - Acceleration

Acceleration mode	GRAPPA
Reference Scans	Integrated
Acceleration Factor PE	3

Resolution - Acceleration

Reference Lines PE	24
Acceleration Factor 3D	1
Phase Partial Fourier	6/8
Slice Partial Fourier	6/8
Asymmetric Echo	Weak

Resolution - Filter

Raw Filter	Off
Elliptical Filter	Off
Distortion Correction	3D
Normalize	Prescan
Image Filter	Off

Geometry - Common

Slab Group	1
Slabs	1
Distance Factor	20 %
Position	Isocenter
Orientation	Sagittal
Phase Encoding Dir.	A >> P
Slices per Slab	128
Phase Oversampling	0 %
Slice Oversampling	0.0 %
FoV Read	260 mm
FoV Phase	100.0 %
Slice Thickness	1.6 mm
TR	3.2 ms
Multi-Slice Mode	Sequential
Series	Ascending
Concatenations	1

Geometry - AutoAlign

Slab Group	1
Position	Isocenter
Orientation	Sagittal
Phase Encoding Dir.	A >> P
AutoAlign	Head
Initial Position	Isocenter
L	0.0 mm
P	0.0 mm
H	0.0 mm
Initial Orientation	Transversal
Initial Rotation	0.00 deg

Geometry - Tim Planning Suite

Set-n-Go Protocol	Off
Table Position	0 mm
Table Position	H
Inline Composing	Off

System - Miscellaneous

Coil Selection	Auto Coil Select
MSMA	S - C - T
Sagittal	R >> L
Coronal	A >> P
Transversal	F >> H
Coil Combination	Adaptive Combine
Matrix Optimization	Off

System - Adjustments

Adjustment Strategy	Standard
B0 Shim	Tune up
B1 Shim	TrueForm
CoilShim	Off
Adjustment Tolerance	Auto
Adjust with Body Coil	Off
Confirm Frequency	Never
Assume Silicone	Off

System - Adjust Volume

Position	Isocenter
Orientation	Transversal
Rotation	0.00 deg
A >> P	263 mm
R >> L	350 mm
F >> H	350 mm
Reset	Off

System - pTx

B1 Shim	TrueForm
Excitation	Non-sel.

System - Tx/Rx

Frequency 1H	123.257785 MHz
? Ref. Amplitude 1H	0.000 V
Reset	Off
Correction Factor	1.00
Image Scaling	1.000

Physio - PACE

Resp. Control	Off
Concatenations	1

Inline - Dynamic

Dynamic Mode	Standard
Flip Angle	8 deg
Measurements	1
Time to Center	6.2 s

Inline - Subtraction

Subtract	Off
Measurements	1
StdDev	Off
Save Original Images	On

Inline - MIP

MIP Sag	Off
MIP Cor	Off
MIP Tra	Off
MIP Time	Off
Radial MIP	Off
Save Original Images	On
MPR Sag	Off
MPR Cor	Off
MPR Tra	Off

Inline - Composing

Inline Composing	Off

Inline - MapIt

MapIt	None
Flip Angle	8 deg
Measurements	1
Contrasts	1
TE	1.37 ms
TR	3.2 ms
Save Original Images	On

Sequence - Part 1

Sequence Name	fl
Dimension	3D
Excitation	Non-sel.
RF Pulse Type	Fast
Gradient Mode	Normal
Bandwidth	540 Hz/Px
Asymmetric Echo	Weak

Sequence - Part 2

Introduction	On
RF Spoiling	On

Sequence - Assistant

SAR Assistant	Off

\\NEURO\HEAD_64\Standard\BIGWIG\t2_space_dark-fluid_sag_iso
TA: 4:32 min Coil Selection: Auto Voxel Size: 0.9×0.9×0.9 mm³ Acc:: 2 Rel. SNR: 1.00

Properties

Start measurement without further preparation	On
Wait for User to Start	Off
Start measurements	Single Measurement
Prio Recon	Off
Auto Open Inline Display	Off
Auto Close Inline Display	Off
Load Images to MR View&GO	On
Auto Store Images	On
Load Images to Stamp Segments	Off
Load Images to Graphic Segments	On
Graphic segment	1st Segment
Inline Movie	Off

Routine

Slab Group	1
Slabs	1
Position	Isocenter
Orientation	Sagittal
Phase Encoding Dir.	P >> A
Slices per Slab	176
Phase Oversampling	0 %
Slice Oversampling	0.0 %
FoV Read	240 mm
FoV Phase	100.0 %
Slice Thickness	0.90 mm
TR	5000.0 ms
TE	398.00 ms
Averages	1.0
Concatenations	1
AutoAlign	Head > Basis
Coil Elements	HC1-7;NC1,2

Contrast - Common

TR	5000.0 ms
TE	398.00 ms
MTC	On
Magn. Preparation	Non-sel. T2-IR
TI 1	1800 ms
Flip Angle Mode	T2 Var
Fat-Water Contrast	Standard
Dark Blood	Off
Blood Suppression	Off
Wrap-up Magn.	None
Reconstruction	Magnitude

Contrast - Dynamic

Dynamic Mode	Standard
Measurements	1
Multiple Series	Each Measurement
Reordering	Linear

Resolution - Common

FoV Read	240 mm
FoV Phase	100.0 %
Slice Thickness	0.90 mm
Base Resolution	256
Phase Resolution	100 %
Slice Resolution	81 %
Interpolation	Off

Resolution - Acceleration

Acceleration mode	GRAPPA
Total Factor	2
Reference Scans	Integrated
Acceleration Factor PE	2
Reference Lines PE	24
Acceleration Factor 3D	1
Phase Partial Fourier	Allowed
Slice Partial Fourier	7/8
Elliptical Scanning	On

Resolution - Filter

Raw Filter	On
Elliptical Filter	Off
Distortion Correction	2D
Normalize	Prescan
Image Filter	Off

Geometry - Common

Slab Group	1
Slabs	1
Position	Isocenter
Orientation	Sagittal
Phase Encoding Dir.	P >> A
Slices per Slab	176
Phase Oversampling	0 %
Slice Oversampling	0.0 %
FoV Read	240 mm
FoV Phase	100.0 %
Slice Thickness	0.90 mm
TR	5000.0 ms
Concatenations	1

Geometry - AutoAlign

Slab Group	1
Position	Isocenter
Orientation	Sagittal
Phase Encoding Dir.	P >> A
AutoAlign	Head > Basis
Initial Position	L0.0 P0.0 H13.0
L	0.0 mm
P	0.0 mm
H	13.0 mm
Initial Orientation	Sagittal
Initial Rotation	-179.94 deg

Geometry - Navigator

Geometry - Saturation

Special Saturation	None

Geometry - Tim Planning Suite

Set-n-Go Protocol	Off
Table Position	13 mm
Table Position	H
Inline Composing	Off

System - Miscellaneous

Coil Selection	Auto Coil Select
MSMA	S - C - T
Sagittal	L >> R

System - Miscellaneous

Coronal	A >> P
Transversal	H >> F
Coil Combination	Adaptive Combine
Matrix Optimization	Off

System - Adjustments

Adjustment Strategy	Standard
B0 Shim	Standard
B1 Shim	TrueForm
CoilShim	Off
Adjustment Tolerance	Auto
Adjust with Body Coil	Off
Confirm Frequency	Never
Assume Silicone	Off

System - Adjust Volume

Position	Isocenter
Orientation	Sagittal
Rotation	-179.94 deg
A >> P	240 mm
F >> H	240 mm
R >> L	159 mm
Reset	Off

System - pTx

B1 Shim	TrueForm
Excitation	Non-sel.

System - Tx/Rx

Frequency 1H	123.257785 MHz
? Ref. Amplitude 1H	0.000 V
Reset	Off
Correction Factor	1.00
Image Scaling	1.000

Physio - Signal

1st Signal/Mode	None
Trigger Delay	0 ms
TR	5000.0 ms
Concatenations	1

Physio - Cardiac

Fat-Water Contrast	Standard
Magn. Preparation	Non-sel. T2-IR
TI 1	1800 ms
Dark Blood	Off
FoV Read	240 mm
FoV Phase	100.0 %
Phase Resolution	100 %
Dynamic Mode	Standard

Physio - PACE

Resp. Control	Off
Concatenations	1

Inline - Subtraction

Subtract	Off
Measurements	1
StdDev	Off
Save Original Images	On

Inline - Cardiac

Magn. Preparation	Non-sel. T2-IR
Save Original Images	On
TE	398.00 ms
TR	5000.0 ms

Inline - MIP

MIP Sag	Off
MIP Cor	Off
MIP Tra	Off
MIP Time	Off
Radial MIP	Off
Save Original Images	On
MPR Sag	Off
MPR Cor	Off
MPR Tra	Off

Inline - Composing

Inline Composing	Off

Sequence - Part 1

Sequence Name	spcir
Dimension	3D
Excitation	Non-sel.
RF Pulse Type	Normal
Gradient Mode	Fast
Flow Compensation	None
Reordering	Linear
Bandwidth	781 Hz/Px
Echo Spacing	3.52 ms
Turbo Factor	284
Echo Train Duration	894 ms

Sequence - Part 2

Introduction	On

Sequence - Assistant

SAR Assistant	Off
Allowed Delay	30 s

\\NEURO\HEAD_64\Standard\BIGWIG\t2_space_sag_iso_SMS2_2rpt_ThuGut_BT#6

TA: 4:57 min Coil Selection: Auto Voxel Size: 0.9×0.9×1.0 mm³ Acc:: 4 Rel. SNR: 1.00

Properties

Start measurement without further preparation	On
Wait for User to Start	Off
Start measurements	Single Measurement
Prio Recon	Off
Auto Open Inline Display	Off
Auto Close Inline Display	Off
Load Images to MR View&GO	On
Auto Store Images	On
Load Images to Stamp Segments	Off
Load Images to Graphic Segments	On
Graphic segment	1st Segment
Inline Movie	Off

Routine

Slab Group	1
Slabs	1
Position	Isocenter
Orientation	Sagittal
Phase Encoding Dir.	P >> A
Slices per Slab	192
Phase Oversampling	0 %
Slice Oversampling	0.0 %
FoV Read	240 mm
FoV Phase	100.0 %
Slice Thickness	1.00 mm
TR	5000.0 ms
TE	398.00 ms
Averages	2.0
Concatenations	1
AutoAlign	Head > Basis
Coil Elements	HE1-4

Contrast - Common

TR	5000.0 ms
TE	398.00 ms
MTC	On
Magn. Preparation	None
Flip Angle Mode	T2 Var
Fat-Water Contrast	Standard
Dark Blood	Off
Blood Suppression	Off
Wrap-up Magn.	None
Reconstruction	Magnitude

Contrast - Dynamic

Dynamic Mode	Standard
Measurements	1
Multiple Series	Each Measurement
Reordering	Linear

Resolution - Common

FoV Read	240 mm
FoV Phase	100.0 %
Slice Thickness	1.00 mm
Base Resolution	256
Phase Resolution	100 %
Slice Resolution	80 %
Interpolation	Off

Resolution - Acceleration

Acceleration mode	CAIPIRINHA
Total Factor	4
Reference Scans	Integrated
Acceleration Factor PE	2
Reference Lines PE	24
Acceleration Factor 3D	2
Reference Lines 3D	24
Reordering Shift 3D	0
Phase Partial Fourier	Allowed
Slice Partial Fourier	Off
Elliptical Scanning	On

Resolution - Filter

Raw Filter	On
Elliptical Filter	Off
Distortion Correction	2D
Normalize	Prescan
Image Filter	Off

Geometry - Common

Slab Group	1
Slabs	1
Position	Isocenter
Orientation	Sagittal
Phase Encoding Dir.	P >> A
Slices per Slab	192
Phase Oversampling	0 %
Slice Oversampling	0.0 %
FoV Read	240 mm
FoV Phase	100.0 %
Slice Thickness	1.00 mm
TR	5000.0 ms
Concatenations	1

Geometry - AutoAlign

Slab Group	1
Position	Isocenter
Orientation	Sagittal
Phase Encoding Dir.	P >> A
AutoAlign	Head > Basis
Initial Position	Isocenter
L	0.0 mm
P	0.0 mm
H	0.0 mm
Initial Orientation	Sagittal
Initial Rotation	-180.00 deg

Geometry - Navigator

Geometry - Saturation

Special Saturation	None

Geometry - Tim Planning Suite

Set-n-Go Protocol	Off
Table Position	0 mm
Table Position	H
Inline Composing	Off

System - Miscellaneous

Coil Selection	Auto Coil Select

System - Miscellaneous

MSMA	S - C - T
Sagittal	R >> L
Coronal	A >> P
Transversal	F >> H
Coil Combination	Adaptive Combine
Matrix Optimization	Off

System - Adjustments

Adjustment Strategy	Standard
B0 Shim	Standard
B1 Shim	TrueForm
CoilShim	Off
Adjustment Tolerance	Auto
Adjust with Body Coil	Off
Confirm Frequency	Never
Assume Silicone	Off

System - Adjust Volume

Position	Isocenter
Orientation	Sagittal
Rotation	180.00 deg
A >> P	240 mm
F >> H	240 mm
R >> L	192 mm
Reset	Off

System - pTx

B1 Shim	TrueForm
Excitation	Non-sel.

System - Tx/Rx

Frequency 1H	123.257785 MHz
? Ref. Amplitude 1H	0.000 V
Reset	Off
Correction Factor	1.00
Image Scaling	1.000

Physio - Signal

1st Signal/Mode	None
Trigger Delay	0 ms
TR	5000.0 ms
Concatenations	1

Physio - Cardiac

Fat-Water Contrast	Standard
Magn. Preparation	None
Dark Blood	Off
FoV Read	240 mm
FoV Phase	100.0 %
Phase Resolution	100 %
Dynamic Mode	Standard

Physio - PACE

Resp. Control	Off
Concatenations	1

Inline - Subtraction

Subtract	Off
Measurements	1
StdDev	Off
Save Original Images	On

Inline - Cardiac

Magn. Preparation	None
Save Original Images	On
TE	398.00 ms
TR	5000.0 ms

Inline - MIP

MIP Sag	Off
MIP Cor	Off
MIP Tra	Off
MIP Time	Off
Radial MIP	Off
Save Original Images	On
MPR Sag	Off
MPR Cor	Off
MPR Tra	Off

Inline - Composing

Inline Composing	Off

Sequence - Part 1

Sequence Name	spc
Dimension	3D
Excitation	Non-sel.
RF Pulse Type	Normal
Gradient Mode	Fast
Flow Compensation	None
Reordering	Linear
Bandwidth	781 Hz/Px
Echo Spacing	3.52 ms
Turbo Factor	284
Echo Train Duration	894 ms

Sequence - Part 2

Introduction	On

Sequence - Assistant

SAR Assistant	Off
Allowed Delay	30 s

\\NEURO\HEAD_64\Standard\BIGWIG\3DT1_sag
TA: 4:19 min Coil Selection: Auto Voxel Size: 1.0×1.0×1.0 mm³ Acc:: 2 Rel. SNR: 1.00

Properties

Start measurement without further preparation	On
Wait for User to Start	Off
Start measurements	Single Measurement
Prio Recon	Off
Auto Open Inline Display	Off
Auto Close Inline Display	Off
Load Images to MR View&GO	On
Auto Store Images	On
Load Images to Stamp Segments	Off
Load Images to Graphic Segments	Off
Graphic segment	Default
Inline Movie	Off

Routine

Slab Group	1
Slabs	1
Distance Factor	50 %
Position	Isocenter
Orientation	Sagittal
Phase Encoding Dir.	A >> P
Slices per Slab	176
Phase Oversampling	0 %
Slice Oversampling	18.2 %
FoV Read	256 mm
FoV Phase	100.0 %
Slice Thickness	1.0 mm
TR	1800.0 ms
TE	2.92 ms
Averages	1
Concatenations	1
AutoAlign	Head > Basis
Coil Elements	HC1-7;NC1,2

Contrast - Common

TR	1800.0 ms
TE	2.92 ms
Magn. Preparation	Non-sel. IR
TI	900 ms
Flip Angle	10 deg
Fat-Water Contrast	Standard
Dark Blood	Off
Reconstruction	Magnitude

Contrast - Dynamic

Dynamic Mode	Standard
Measurements	1
Multiple Series	Off
Reordering	Linear

Resolution - Common

FoV Read	256 mm
FoV Phase	100.0 %
Slice Thickness	1.0 mm
Base Resolution	256
Phase Resolution	100 %
Slice Resolution	100 %
Interpolation	Off

Resolution - Acceleration

Acceleration mode	GRAPPA
Reference Scans	Integrated
Acceleration Factor PE	2
Reference Lines PE	32
Acceleration Factor 3D	1
Phase Partial Fourier	Off
Slice Partial Fourier	Off
Asymmetric Echo	Off
Elliptical Scanning	Off

Resolution - Filter

Raw Filter	Off
Elliptical Filter	Off
Distortion Correction	3D
Normalize	Prescan
Image Filter	Off

Geometry - Common

Slab Group	1
Slabs	1
Distance Factor	50 %
Position	Isocenter
Orientation	Sagittal
Phase Encoding Dir.	A >> P
Slices per Slab	176
Phase Oversampling	0 %
Slice Oversampling	18.2 %
FoV Read	256 mm
FoV Phase	100.0 %
Slice Thickness	1.0 mm
TR	1800.0 ms
Multi-Slice Mode	Single Shot
Series	Interleaved
Concatenations	1

Geometry - AutoAlign

Slab Group	1
Position	Isocenter
Orientation	Sagittal
Phase Encoding Dir.	A >> P
AutoAlign	Head > Basis
Initial Position	Isocenter
L	0.0 mm
A	0.0 mm
H	0.0 mm
Initial Orientation	Sagittal
Initial Rotation	0.01 deg

Geometry - Navigator

Geometry - Tim Planning Suite

Set-n-Go Protocol	Off
Table Position	0 mm
Table Position	H
Inline Composing	Off

System - Miscellaneous

Coil Selection	Auto Coil Select
MSMA	S - C - T
Sagittal	R >> L

System - Miscellaneous

Coronal	A >> P
Transversal	F >> H
Coil Combination	Adaptive Combine
Matrix Optimization	Off

System - Adjustments

Adjustment Strategy	Standard
B0 Shim	Standard
B1 Shim	TrueForm
CoilShim	Off
Adjustment Tolerance	Auto
Adjust with Body Coil	Off
Confirm Frequency	Never
Assume Silicone	Off

System - Adjust Volume

! Position	L0.0 P11.2 H0.0 mm
! Orientation	Sagittal
! Rotation	0.01 deg
! A >> P	205 mm
! F >> H	256 mm
! R >> L	160 mm
Reset	Off

System - pTx

B1 Shim	TrueForm
Excitation	Non-sel.

System - Tx/Rx

Frequency 1H	123.257785 MHz
? Ref. Amplitude 1H	0.000 V
Reset	Off
Correction Factor	1.00
Image Scaling	1.000

Physio - Signal

1st Signal/Mode	None
TR	1800.0 ms
Concatenations	1

Physio - Cardiac

Fat-Water Contrast	Standard
Magn. Preparation	Non-sel. IR
TI	900 ms
Dark Blood	Off
FoV Read	256 mm
FoV Phase	100.0 %
Phase Resolution	100 %
Dynamic Mode	Standard

Physio - PACE

Resp. Control	Off
Concatenations	1

Inline - Subtraction

Subtract	On
Subtraction Mode	Standard
Save Subtracted Images	On
Subtrahend	1
Subtraction Group	1
Measurements	1
Autoscaling	On
StdDev	Off

Inline - Subtraction

Save Original Images	On

Inline - Cardiac

Magn. Preparation	Non-sel. IR
Save Original Images	On
TE	2.92 ms
TR	1800.0 ms

Inline - MIP

MIP Sag	Off
MIP Cor	Off
MIP Tra	Off
MIP Time	Off
Radial MIP	Off
Save Original Images	On
MPR Sag	Off
MPR Cor	Off
MPR Tra	Off

Inline - Composing

Inline Composing	Off

Inline - MapIt

MapIt	None
Flip Angle	10 deg
Measurements	1
TE	2.92 ms
TR	1800.0 ms
Save Original Images	On

Sequence - Part 1

Sequence Name	tfl
Dimension	3D
Excitation	Non-sel.
RF Pulse Type	Fast
Gradient Mode	Performance
Flow Compensation	None
Reordering	Linear
Bandwidth	240 Hz/Px
Echo Spacing	6.76 ms
Asymmetric Echo	Off
Turbo Factor	208

Sequence - Part 2

Introduction	On
RF Spoiling	On
Incr. Gradient Spoiling	Off
BM Motion Correction	Off

Sequence - Assistant

SAR Assistant	Off

\\NEURO\HEAD_64\Standard\BIGWIG\SWI_MIU
TA: 7:39 min Coil Selection: Auto Voxel Size: 0.3×0.3×1.5 mm³ Acc:: None Rel. SNR: 1.00

Properties

Start measurement without further preparation	On
Wait for User to Start	Off
Start measurements	Single Measurement
Prio Recon	Off
Auto Open Inline Display	Off
Auto Close Inline Display	Off
Load Images to MR View&GO	On
Auto Store Images	On
Load Images to Stamp Segments	Off
Load Images to Graphic Segments	Off
Graphic segment	Default
Inline Movie	Off

Routine

Slab Group	1
Slabs	1
Distance Factor	20 %
Position	Isocenter
Orientation	Transversal
Phase Encoding Dir.	R >> L
Slices per Slab	88
Phase Oversampling	0 %
Slice Oversampling	0.0 %
FoV Read	220 mm
FoV Phase	68.8 %
Slice Thickness	1.5 mm
TR	35.0 ms
TE 1	7.67 ms
TE 2	24.60 ms
Averages	1
Concatenations	1
AutoAlign	Head > Brain
Coil Elements	HC1-7

Contrast - Common

TR	35.0 ms
TE 1	7.67 ms
TE 2	24.60 ms
MTC	Off
Magn. Preparation	None
Flip Angle	20 deg
Fat-Water Contrast	Standard
Dark Blood	Off
Contrasts	2
SWI	Off
Reconstruction	Magn./Phase

Contrast - Dynamic

Dynamic Mode	Standard
Measurements	1
Multiple Series	Each Measurement

Resolution - Common

FoV Read	220 mm
FoV Phase	68.8 %
Slice Thickness	1.5 mm
Base Resolution	384
Phase Resolution	100 %
Slice Resolution	100 %

Resolution - Common

Interpolation	On

Resolution - Acceleration

Acceleration mode	None
Phase Partial Fourier	6/8
Slice Partial Fourier	6/8
Asymmetric Echo	Off
Elliptical Scanning	Off

Resolution - Filter

Raw Filter	Off
Elliptical Filter	On
Distortion Correction	2D
Normalize	Prescan
Image Filter	Off

Geometry - Common

Slab Group	1
Slabs	1
Distance Factor	20 %
Position	Isocenter
Orientation	Transversal
Phase Encoding Dir.	R >> L
Slices per Slab	88
Phase Oversampling	0 %
Slice Oversampling	0.0 %
FoV Read	220 mm
FoV Phase	68.8 %
Slice Thickness	1.5 mm
TR	35.0 ms
Multi-Slice Mode	Interleaved
Series	Interleaved
Concatenations	1

Geometry - AutoAlign

Slab Group	1
Position	Isocenter
Orientation	Transversal
Phase Encoding Dir.	R >> L
AutoAlign	Head > Brain
Initial Position	Isocenter
R	0.0 mm
P	0.0 mm
F	0.0 mm
Initial Orientation	Transversal
Initial Rotation	90.00 deg

Geometry - Saturation

Saturation Mode	Standard
Special Saturation	None

Geometry - Tim Planning Suite

Set-n-Go Protocol	Off
Table Position	0 mm
Table Position	H
Inline Composing	Off

System - Miscellaneous

Coil Selection	Auto Coil Select
MSMA	S - C - T

System - Miscellaneous

Sagittal	R >> L
Coronal	A >> P
Transversal	F >> H
Coil Combination	Adaptive Combine
Matrix Optimization	Off

System - Adjustments

Adjustment Strategy	Standard
B0 Shim	Standard
B1 Shim	TrueForm
CoilShim	Off
Adjustment Tolerance	Auto
Adjust with Body Coil	Off
Confirm Frequency	Never
Assume Silicone	Off

System - Adjust Volume

! Position	Isocenter
! Orientation	Transversal
! Rotation	90.09 deg
! R >> L	152 mm
! A >> P	220 mm
! F >> H	132 mm
Reset	Off

System - pTx

B1 Shim	TrueForm
Excitation	Slab-sel.
LR Balancing	Off

System - Tx/Rx

Frequency 1H	123.257785 MHz
? Ref. Amplitude 1H	0.000 V
Reset	Off
Correction Factor	1.00
Image Scaling	1.000

Physio - Signal

1st Signal/Mode	None
TR	35.0 ms
Segments	1
Concatenations	1

Physio - Cardiac

Tagging	None
Fat-Water Contrast	Standard
Magn. Preparation	None
Dark Blood	Off
FoV Read	220 mm
FoV Phase	68.8 %
Phase Resolution	100 %
Dynamic Mode	Standard

Physio - PACE

Resp. Control	Off
Concatenations	1

Inline - Liver

Liver Registration	Off
Save Original Images	On

Inline - Subtraction

Subtract	Off
Measurements	1
StdDev	Off
Save Original Images	On

Inline - Cardiac

Magn. Preparation	None
Save Original Images	On
Contrasts	2
TE 1	7.67 ms
TE 2	24.60 ms
TR	35.0 ms

Inline - MIP

MIP Sag	Off
MIP Cor	Off
MIP Tra	Off
MIP Time	Off
Radial MIP	Off
Save Original Images	On
MPR Sag	Off
MPR Cor	Off
MPR Tra	Off

Inline - Soft Tissue

Wash-in	Off
Wash-out	Off
TTP	Off
PEI	Off
MIP Time	Off
Measurements	1

Inline - Composing

Inline Composing	Off

Inline - MapIt

MapIt	None
Flip Angle	20 deg
Measurements	1
Contrasts	2
TE 1	7.67 ms
TE 2	24.60 ms
TR	35.0 ms
Save Original Images	On

Sequence - Part 1

Sequence Name	fl_r
Dimension	3D
Excitation	Slab-sel.
RF Pulse Type	Normal
Readout Mode	Monopolar
Gradient Mode	Fast
Flow Compensation 1	Slice/Read
Flow Compensation 2	None
Bandwidth 1	200 Hz/Px
Bandwidth 2	200 Hz/Px
Asymmetric Echo	Off
Segments	1

Sequence - Part 2

Introduction	On
RF Spoiling	On
BM Motion Correction	Off
Acoustic noise reduction	Off

Sequence - Assistant

SAR Assistant	Off
Allowed Delay	0 s

\\NEURO\HEAD_64\Standard\BIGWIG\resolve_diff_224
TA: 3:52 min Coil Selection: Auto Voxel Size: 0.5×0.5×4.0 mm³ Acc:: 2 Rel. SNR: 1.00

Properties

Start measurement without further preparation	On
Wait for User to Start	Off
Start measurements	Single Measurement
Prio Recon	Off
Auto Open Inline Display	Off
Auto Close Inline Display	Off
Load Images to MR View&GO	On
Auto Store Images	On
Load Images to Stamp Segments	On
Load Images to Graphic Segments	On
Graphic segment	3rd Segment
Inline Movie	Off

Routine

Slice Group	1
Slices	27
Distance Factor	30 %
Position	Isocenter
Orientation	Transversal
Phase Encoding Dir.	A >> P
Phase Oversampling	0 %
FoV Read	220 mm
FoV Phase	100.0 %
Slice Thickness	4.0 mm
TR	5910.0 ms
TE 1	66.00 ms
TE 2	114.00 ms
Concatenations	1
AutoAlign	---
Coil Elements	HC1-7

Contrast - Common

TR	5910.0 ms
TE 1	66.00 ms
TE 2	114.00 ms
MTC	Off
Magn. Preparation	None
Flip Angle	180 deg
Fat-Water Contrast	Fat Saturation
Fat Saturation	Strong
Contrasts	2
Reconstruction	Magnitude

Contrast - Dynamic

Dynamic Mode	Standard
Measurements	1

Resolution - Common

FoV Read	220 mm
FoV Phase	100.0 %
Slice Thickness	4.0 mm
Base Resolution	224
Phase Resolution	100 %
Interpolation	On

Resolution - Acceleration

Accel. Mode	GRAPPA
Reference Scans	EPI/Separate
Acceleration Factor PE	2

Resolution - Acceleration

Reference Lines PE	112
Phase Partial Fourier	Off
Readout Partial Fourier	7/8
Readout Segments	7

Resolution - Filter

Raw Filter	On
Distortion Correction	2D
Normalize	Prescan

Geometry - Common

Slice Group	1
Slices	27
Distance Factor	30 %
Position	Isocenter
Orientation	Transversal
Phase Encoding Dir.	A >> P
Phase Oversampling	0 %
FoV Read	220 mm
FoV Phase	100.0 %
Slice Thickness	4.0 mm
TR	5910.0 ms
Multi-Slice Mode	Interleaved
Series	Interleaved
Concatenations	1

Geometry - AutoAlign

Slice Group	1
Position	Isocenter
Orientation	Transversal
Phase Encoding Dir.	A >> P
AutoAlign	---
Initial Position	Isocenter
L	0.0 mm
P	0.0 mm
H	0.0 mm
Initial Orientation	Transversal
Initial Rotation	0.00 deg

Geometry - Saturation

Special Saturation	None

Geometry - Tim Planning Suite

Set-n-Go Protocol	Off
Table Position	0 mm
Table Position	H
Inline Composing	Off

System - Miscellaneous

Coil Selection	Auto Coil Select
MSMA	S - C - T
Sagittal	L >> R
Coronal	A >> P
Transversal	H >> F
Coil Combination	Adaptive Combine
Matrix Optimization	Off

System - Adjustments

Adjustment Strategy	Standard
B0 Shim	Standard

System - Adjustments

B1 Shim	TrueForm
CoilShim	Off
Adjustment Tolerance	Auto
Adjust with Body Coil	Off
Confirm Frequency	Never
Assume Silicone	Off

System - Adjust Volume

Position	Isocenter
Orientation	Transversal
Rotation	0.00 deg
A >> P	220 mm
R >> L	220 mm
F >> H	140 mm
Reset	Off

System - pTx

B1 Shim	TrueForm

System - Tx/Rx

Frequency 1H	123.257785 MHz
? Ref. Amplitude 1H	0.000 V
Reset	Off
Correction Factor	1.00
Image Scaling	1.000

Physio - Signal

1st Signal/Mode	None
TR	5910.0 ms
Concatenations	1

Diff

Diffusion Mode	4-Scan Trace
Diff. Directions	4
Diffusion Scheme	Monopolar
Diff. Weightings	2
b-value 1	0 s/mm²
b-value 2	1000 s/mm²
Averages 1	1
Averages 2	1
Invert Gray Scale	Off
Diff. Weighted Images	Off
Trace Weighted Images	On
Tensor	Off
FA Maps	Off
ADC Maps	On
Exponential ADC Maps	Off
b-value >=	0 s/mm²
ADC Noise Threshold	100
Noise Masking	Off
Calculated Image	Off

Sequence - Part 1

Sequence Name	resolve
Dimension	2D
RF Pulse Type	Normal
Gradient Mode	Fast
Bandwidth	657 Hz/Px
Echo Spacing	0.36 ms
Optimization	Min. TE
EPI Factor	112

Sequence - Part 2

Introduction	On
Reacquisition Mode	On

Sequence - Assistant

SAR Assistant	Off
Optimization	Min. TE

\\NEURO\HEAD_64\Standard\BIGWIG\AAspine_scout

TA: 19 sec Coil Selection: Auto Voxel Size: 1.7×1.7×1.7 mm³ Acc:: 3 Rel. SNR: 1.00

Properties

Start measurement without further preparation	Off
Wait for User to Start	Off
Start measurements	Single Measurement
Prio Recon	Off
Auto Open Inline Display	Off
Auto Close Inline Display	Off
Load Images to MR View&GO	On
Auto Store Images	On
Load Images to Stamp Segments	On
Load Images to Graphic Segments	On
Graphic segment	Default
Inline Movie	Off

Routine

Slab Group	1
Slabs	1
Distance Factor	20 %
Position	L0.0 P20.0 H0.0 mm
Orientation	Coronal
Phase Encoding Dir.	F >> H
Slices per Slab	96
Phase Oversampling	10 %
Slice Oversampling	25.0 %
FoV Read	400 mm
FoV Phase	100.0 %
Slice Thickness	1.7 mm
TR	3.3 ms
TE	1.27 ms
Averages	1
Concatenations	1
AutoAlign	Spine
Coil Elements	HC6,7;NC1,2;S1,2

Contrast - Common

TR	3.3 ms
TE	1.27 ms
Flip Angle	8 deg
Fat-Water Contrast	Standard
Contrasts	1
Reconstruction	Magnitude

Contrast - Dynamic

Dynamic Mode	Standard
Measurements	1
Time to Center	8.4 s

Resolution - Common

FoV Read	400 mm
FoV Phase	100.0 %
Slice Thickness	1.7 mm
Base Resolution	240
Phase Resolution	100 %
Slice Resolution	68 %
Trajectory	Cartesian

Resolution - Acceleration

Acceleration mode	GRAPPA
Reference Scans	Integrated
Acceleration Factor PE	3

Resolution - Acceleration

Reference Lines PE	24
Acceleration Factor 3D	1
Phase Partial Fourier	6/8
Slice Partial Fourier	6/8
Asymmetric Echo	Weak

Resolution - Filter

Raw Filter	Off
Elliptical Filter	Off
Distortion Correction	3D
Normalize	Prescan
Image Filter	Off

Geometry - Common

Slab Group	1
Slabs	1
Distance Factor	20 %
Position	L0.0 P20.0 H0.0 mm
Orientation	Coronal
Phase Encoding Dir.	F >> H
Slices per Slab	96
Phase Oversampling	10 %
Slice Oversampling	25.0 %
FoV Read	400 mm
FoV Phase	100.0 %
Slice Thickness	1.7 mm
TR	3.3 ms
Multi-Slice Mode	Sequential
Series	Ascending
Concatenations	1

Geometry - AutoAlign

Slab Group	1
Position	L0.0 P20.0 H0.0 mm
Orientation	Coronal
Phase Encoding Dir.	F >> H
AutoAlign	Spine
Initial Position	Isocenter
L	0.0 mm
P	0.0 mm
H	0.0 mm
Initial Orientation	Transversal
Initial Rotation	0.00 deg

Geometry - Tim Planning Suite

Set-n-Go Protocol	Off
Table Position	0 mm
Table Position	H
Inline Composing	Off

System - Miscellaneous

Coil Selection	Auto Coil Select
MSMA	S - C - T
Sagittal	L >> R
Coronal	A >> P
Transversal	F >> H
Coil Combination	Adaptive Combine
Matrix Optimization	Off
Coil Focus	Center

System - Adjustments

Adjustment Strategy	Standard
B0 Shim	Tune up
B1 Shim	TrueForm
CoilShim	Off
Adjustment Tolerance	Auto
Adjust with Body Coil	Off
Confirm Frequency	Never
Assume Silicone	Off

System - Adjust Volume

Position	Isocenter
Orientation	Transversal
Rotation	0.00 deg
A >> P	263 mm
R >> L	350 mm
F >> H	350 mm
Reset	Off

System - pTx

B1 Shim	TrueForm
Excitation	Non-sel.

System - Tx/Rx

Frequency 1H	123.257785 MHz
? Ref. Amplitude 1H	0.000 V
Reset	Off
Correction Factor	1.00
Image Scaling	1.000

Physio - PACE

Resp. Control	Off
Concatenations	1

Inline - Dynamic

Dynamic Mode	Standard
Flip Angle	8 deg
Measurements	1
Time to Center	8.4 s

Inline - Subtraction

Subtract	Off
Measurements	1
StdDev	Off
Save Original Images	On

Inline - MIP

MIP Sag	Off
MIP Cor	Off
MIP Tra	Off
MIP Time	Off
Radial MIP	Off
Save Original Images	On
MPR Sag	Off
MPR Cor	Off
MPR Tra	Off

Inline - Composing

Inline Composing	Off

Inline - MapIt

MapIt	None
Flip Angle	8 deg
Measurements	1
Contrasts	1
TE	1.27 ms
TR	3.3 ms
Save Original Images	On

Sequence - Part 1

Sequence Name	fl
Dimension	3D
Excitation	Non-sel.
RF Pulse Type	Fast
Gradient Mode	Normal
Bandwidth	550 Hz/Px
Asymmetric Echo	Weak

Sequence - Part 2

Introduction	On
RF Spoiling	On

Sequence - Assistant

SAR Assistant	Off

\\NEURO\HEAD_64\Standard\BIGWIG\t2_tse_sag_HWS
TA: 1:47 min Coil Selection: Auto Voxel Size: 0.6×0.6×3.0 mm³ Acc:: 2 Rel. SNR: 1.00

Properties

Start measurement without further preparation	On
Wait for User to Start	Off
Start measurements	Single Measurement
Prio Recon	Off
Auto Open Inline Display	Off
Auto Close Inline Display	Off
Load Images to MR View&GO	On
Auto Store Images	On
Load Images to Stamp Segments	On
Load Images to Graphic Segments	Off
Graphic segment	Default
Inline Movie	Off

Routine

Slice Group	1
Slices	15
Distance Factor	10 %
Position	L0.0 P20.0 H0.0 mm
Orientation	Sagittal
Phase Encoding Dir.	H >> F
Phase Oversampling	80 %
FoV Read	220 mm
FoV Phase	100.0 %
Slice Thickness	3.0 mm
TR	3500.0 ms
TE	106.00 ms
Averages	1
Concatenations	2
AutoAlign	Spine > Cervical
Coil Elements	HC6,7;NC1,2;S1

Contrast - Common

TR	3500.0 ms
TE	106.00 ms
TD	0.00 ms
MTC	Off
Magn. Preparation	None
Flip Angle	160 deg
Fat-Water Contrast	Standard
Dark Blood	Off
Contrasts	1
Wrap-up Magn.	Restore
Reconstruction	Magnitude

Contrast - Dynamic

Dynamic Mode	Standard
Measurements	1
Multiple Series	Each Measurement

Resolution - Common

FoV Read	220 mm
FoV Phase	100.0 %
Slice Thickness	3.0 mm
Base Resolution	384
Phase Resolution	70 %
Trajectory	Cartesian
Interpolation	Off

Resolution - Acceleration

Acceleration mode	GRAPPA
Reference Scans	Integrated
Acceleration Factor PE	2
Reference Lines PE	47
Phase Partial Fourier	Off

Resolution - Filter

Raw Filter	Off
Elliptical Filter	On
Distortion Correction	2D
Normalize	Prescan
Image Filter	On

Geometry - Common

Slice Group	1
Slices	15
Distance Factor	10 %
Position	L0.0 P20.0 H0.0 mm
Orientation	Sagittal
Phase Encoding Dir.	H >> F
Phase Oversampling	80 %
FoV Read	220 mm
FoV Phase	100.0 %
Slice Thickness	3.0 mm
TR	3500.0 ms
Multi-Slice Mode	Interleaved
Series	Interleaved
Concatenations	2

Geometry - AutoAlign

Slice Group	1
Position	L0.0 P20.0 H0.0 mm
Orientation	Sagittal
Phase Encoding Dir.	H >> F
AutoAlign	Spine > Cervical
Initial Position	L0.0 P20.0 H0.0
L	0.0 mm
P	20.0 mm
H	0.0 mm
Initial Orientation	Sagittal
Initial Rotation	90.00 deg

Geometry - Navigator

Geometry - Saturation

Saturation Region	1
Thickness	80.00 mm
Position	L0.0 A60.0 H0.0 mm
Orientation	Coronal
Shape	Standard
Special Saturation	None

Geometry - Tim Planning Suite

Set-n-Go Protocol	Off
Table Position	0 mm
Table Position	H
Inline Composing	Off

System - Miscellaneous

Coil Selection	Auto Coil Select

System - Miscellaneous

MSMA	S - C - T
Sagittal	L >> R
Coronal	A >> P
Transversal	F >> H
Coil Combination	Adaptive Combine
Matrix Optimization	Off
Coil Focus	Flat

System - Adjustments

Adjustment Strategy	Standard
B0 Shim	Tune up
B1 Shim	TrueForm
CoilShim	Off
Adjustment Tolerance	Auto
Adjust with Body Coil	Off
Confirm Frequency	Never
Assume Silicone	Off

System - Adjust Volume

Position	Isocenter
Orientation	Transversal
Rotation	0.00 deg
A >> P	263 mm
R >> L	350 mm
F >> H	350 mm
Reset	Off

System - pTx

B1 Shim	TrueForm
LR Balancing	Off

System - Tx/Rx

Frequency 1H	123.257785 MHz
? Ref. Amplitude 1H	0.000 V
Reset	Off
Correction Factor	1.00
Image Scaling	1.000

Physio - Signal

1st Signal/Mode	None
TR	3500.0 ms
Concatenations	2

Physio - Cardiac

Fat-Water Contrast	Standard
Magn. Preparation	None
Dark Blood	Off
FoV Read	220 mm
FoV Phase	100.0 %
Phase Resolution	70 %
Trajectory	Cartesian
Dynamic Mode	Standard

Physio - PACE

Resp. Control	Off
Concatenations	2

Inline - Subtraction

Subtract	Off
Measurements	1
StdDev	Off
Save Original Images	On

Inline - Cardiac

Magn. Preparation	None
Save Original Images	On
Contrasts	1
TE	106.00 ms
TR	3500.0 ms

Inline - MIP

MIP Sag	Off
MIP Cor	Off
MIP Tra	Off
MIP Time	Off
Radial MIP	Off
Save Original Images	On
MPR Sag	Off
MPR Cor	Off
MPR Tra	Off

Inline - Composing

Inline Composing	Off

Sequence - Part 1

Sequence Name	tseR_rr
Dimension	2D
RF Pulse Type	Normal
Gradient Mode	Normal
Flow Compensation	Read
Bandwidth	260 Hz/Px
Echo Spacing	9.62 ms
Free Echo Spacing	Off
Define	Turbo Factor
Turbo Factor	19
Echo Trains per Slice	14

Sequence - Part 2

Introduction	On
BM Motion Correction	Off
Phase Correction	Automatic
Compensate T2 Decay	Off
Hyperecho	Off
WARP	Off
Red. EC Sensitivity	Off
Acoustic noise reduction	Off
Reduce Motion Sens.	On

Sequence - Assistant

SAR Assistant	Flip Angle, TR
Min Flip Angle	130 deg
Max. TR	4000.0 ms
Allowed Delay	60 s

\\NEURO\HEAD_64\Standard\BIGWIG\3DT1_tra_KM

TA: 2:55 min Coil Selection: Auto Voxel Size: 0.9×0.9×3.0 mm³ Acc:: 2 Rel. SNR: 1.00

Properties

Start measurement without further preparation	On
Wait for User to Start	Off
Start measurements	Single Measurement
Prio Recon	Off
Auto Open Inline Display	Off
Auto Close Inline Display	Off
Load Images to MR View&GO	On
Auto Store Images	On
Load Images to Stamp Segments	On
Load Images to Graphic Segments	Off
Graphic segment	Default
Inline Movie	Off

Routine

Slab Group	1
Slabs	1
Distance Factor	50 %
Position	Isocenter
Orientation	Transversal
Phase Encoding Dir.	R >> L
Slices per Slab	60
Phase Oversampling	0 %
Slice Oversampling	60.0 %
FoV Read	240 mm
FoV Phase	87.5 %
Slice Thickness	3.0 mm
TR	1410.0 ms
TE	3.30 ms
Averages	1
Concatenations	1
AutoAlign	Head > Brain
Coil Elements	HC1-7

Contrast - Common

TR	1410.0 ms
TE	3.30 ms
Magn. Preparation	Slice-sel. IR
TI	900 ms
Flip Angle	9 deg
Fat-Water Contrast	Standard
Dark Blood	Off
Reconstruction	Magnitude

Contrast - Dynamic

Dynamic Mode	Standard
Measurements	1
Multiple Series	Each Measurement
Reordering	Linear

Resolution - Common

FoV Read	240 mm
FoV Phase	87.5 %
Slice Thickness	3.0 mm
Base Resolution	256
Phase Resolution	100 %
Slice Resolution	100 %
Interpolation	Off

Resolution - Acceleration

Acceleration mode	GRAPPA
Reference Scans	Integrated
Acceleration Factor PE	2
Reference Lines PE	24
Acceleration Factor 3D	1
Phase Partial Fourier	Off
Slice Partial Fourier	Off
Asymmetric Echo	Off
Elliptical Scanning	Off

Resolution - Filter

Raw Filter	Off
Elliptical Filter	On
Distortion Correction	2D
Normalize	Prescan
Image Filter	On

Geometry - Common

Slab Group	1
Slabs	1
Distance Factor	50 %
Position	Isocenter
Orientation	Transversal
Phase Encoding Dir.	R >> L
Slices per Slab	60
Phase Oversampling	0 %
Slice Oversampling	60.0 %
FoV Read	240 mm
FoV Phase	87.5 %
Slice Thickness	3.0 mm
TR	1410.0 ms
Multi-Slice Mode	Single Shot
Series	Ascending
Concatenations	1

Geometry - AutoAlign

Slab Group	1
Position	Isocenter
Orientation	Transversal
Phase Encoding Dir.	R >> L
AutoAlign	Head > Brain
Initial Position	Isocenter
L	0.0 mm
A	0.0 mm
H	0.0 mm
Initial Orientation	Transversal
Initial Rotation	89.93 deg

Geometry - Navigator

Geometry - Tim Planning Suite

Set-n-Go Protocol	Off
Table Position	0 mm
Table Position	H
Inline Composing	Off

System - Miscellaneous

Coil Selection	Auto Coil Select
MSMA	S - C - T
Sagittal	L >> R

System - Miscellaneous

Coronal	A >> P
Transversal	H >> F
Coil Combination	Adaptive Combine
Matrix Optimization	Off

System - Adjustments

Adjustment Strategy	Standard
B0 Shim	Tune up
B1 Shim	TrueForm
CoilShim	Off
Adjustment Tolerance	Auto
Adjust with Body Coil	Off
Confirm Frequency	Never
Assume Silicone	Off

System - Adjust Volume

Position	Isocenter
Orientation	Transversal
Rotation	0.00 deg
A >> P	263 mm
R >> L	350 mm
F >> H	350 mm
Reset	Off

System - pTx

B1 Shim	TrueForm
Excitation	Slab-sel.

System - Tx/Rx

Frequency 1H	123.257785 MHz
? Ref. Amplitude 1H	0.000 V
Reset	Off
Correction Factor	1.00
Image Scaling	1.000

Physio - Signal

1st Signal/Mode	None
TR	1410.0 ms
Concatenations	1

Physio - Cardiac

Fat-Water Contrast	Standard
Magn. Preparation	Slice-sel. IR
TI	900 ms
Dark Blood	Off
FoV Read	240 mm
FoV Phase	87.5 %
Phase Resolution	100 %
Dynamic Mode	Standard

Physio - PACE

Resp. Control	Off
Concatenations	1

Inline - Subtraction

Subtract	Off
Measurements	1
StdDev	Off
Save Original Images	On

Inline - Cardiac

Magn. Preparation	Slice-sel. IR
Save Original Images	On

Inline - Cardiac

TE	3.30 ms
TR	1410.0 ms

Inline - MIP

MIP Sag	Off
MIP Cor	Off
MIP Tra	Off
MIP Time	Off
Radial MIP	Off
Save Original Images	On
MPR Sag	Off
MPR Cor	Off
MPR Tra	Off

Inline - Composing

Inline Composing	Off

Inline - MapIt

MapIt	None
Flip Angle	9 deg
Measurements	1
TE	3.30 ms
TR	1410.0 ms
Save Original Images	On

Sequence - Part 1

Sequence Name	tfl
Dimension	3D
Excitation	Slab-sel.
RF Pulse Type	Fast
Gradient Mode	Performance
Flow Compensation	None
Reordering	Linear
Bandwidth	250 Hz/Px
Echo Spacing	7.66 ms
Asymmetric Echo	Off
Turbo Factor	96

Sequence - Part 2

Introduction	On
RF Spoiling	On
Incr. Gradient Spoiling	On
BM Motion Correction	Off

Sequence - Assistant

SAR Assistant	Off

\\NEURO\HEAD_64\Standard\BIGWIG\T1_space_fs_BB_sag
TA: 4:46 min Coil Selection: Auto Voxel Size: 0.9×0.9×0.9 mm³ Acc:: 4 Rel. SNR: 1.00

Properties

Start measurement without further preparation	On
Wait for User to Start	Off
Start measurements	Single Measurement
Prio Recon	Off
Auto Open Inline Display	Off
Auto Close Inline Display	Off
Load Images to MR View&GO	On
Auto Store Images	On
Load Images to Stamp Segments	Off
Load Images to Graphic Segments	Off
Graphic segment	Default
Inline Movie	Off

Routine

Slab Group	1
Slabs	1
Position	L0.0 P2.9 H4.2 mm
Orientation	Sagittal
Phase Encoding Dir.	A >> P
Slices per Slab	192
Phase Oversampling	10 %
Slice Oversampling	0.0 %
FoV Read	230 mm
FoV Phase	100.0 %
Slice Thickness	0.90 mm
TR	700.0 ms
TE	11.00 ms
Averages	1.4
Concatenations	1
AutoAlign	Head > Basis
Coil Elements	HC1-7

Contrast - Common

TR	700.0 ms
TE	11.00 ms
MTC	Off
Magn. Preparation	None
Flip Angle Mode	T1 Var
Fat-Water Contrast	SPAIR
Fat Saturation	Strong
Dark Blood	Off
Blood Suppression	Free
Wrap-up Magn.	Restore
Reconstruction	Magnitude

Contrast - Dynamic

Dynamic Mode	Standard
Measurements	1
Multiple Series	Each Measurement
Reordering	Radial

Resolution - Common

FoV Read	230 mm
FoV Phase	100.0 %
Slice Thickness	0.90 mm
Base Resolution	256
Phase Resolution	100 %
Slice Resolution	99 %
Interpolation	Off

Resolution - Acceleration

Acceleration mode	GRAPPA
Total Factor	4
Reference Scans	Integrated
Acceleration Factor PE	2
Reference Lines PE	24
Acceleration Factor 3D	2
Reference Lines 3D	24
Phase Partial Fourier	Off
Slice Partial Fourier	Off
Elliptical Scanning	On

Resolution - Filter

Raw Filter	On
Elliptical Filter	Off
Distortion Correction	2D
Normalize	Prescan
Image Filter	Off

Geometry - Common

Slab Group	1
Slabs	1
Position	L0.0 P2.9 H4.2 mm
Orientation	Sagittal
Phase Encoding Dir.	A >> P
Slices per Slab	192
Phase Oversampling	10 %
Slice Oversampling	0.0 %
FoV Read	230 mm
FoV Phase	100.0 %
Slice Thickness	0.90 mm
TR	700.0 ms
Concatenations	1

Geometry - AutoAlign

Slab Group	1
Position	L0.0 P2.9 H4.2 mm
Orientation	Sagittal
Phase Encoding Dir.	A >> P
AutoAlign	Head > Basis
Initial Position	L0.0 P2.9 H4.2
L	0.0 mm
P	2.9 mm
H	4.2 mm
Initial Orientation	Sagittal
Initial Rotation	0.01 deg

Geometry - Navigator

Geometry - Saturation

Special Saturation	None

Geometry - Tim Planning Suite

Set-n-Go Protocol	Off
Table Position	4 mm
Table Position	H
Inline Composing	Off

System - Miscellaneous

Coil Selection	Auto Coil Select
MSMA	S - C - T

System - Miscellaneous

Sagittal	R >> L
Coronal	A >> P
Transversal	F >> H
Coil Combination	Adaptive Combine
Matrix Optimization	Performance

System - Adjustments

Adjustment Strategy	Standard
B0 Shim	Standard
B1 Shim	TrueForm
CoilShim	Off
Adjustment Tolerance	Auto
Adjust with Body Coil	Off
Confirm Frequency	Never
Assume Silicone	Off

System - Adjust Volume

Position	L0.0 P2.9 H4.2 mm
Orientation	Sagittal
Rotation	0.01 deg
A >> P	230 mm
F >> H	230 mm
R >> L	173 mm
Reset	Off

System - pTx

B1 Shim	TrueForm
Excitation	Non-sel.

System - Tx/Rx

Frequency 1H	123.257785 MHz
? Ref. Amplitude 1H	0.000 V
Reset	Off
Correction Factor	1.00
Image Scaling	1.000

Physio - Signal

1st Signal/Mode	None
Trigger Delay	0 ms
TR	700.0 ms
Concatenations	1

Physio - Cardiac

Fat-Water Contrast	SPAIR
Magn. Preparation	None
Dark Blood	Off
FoV Read	230 mm
FoV Phase	100.0 %
Phase Resolution	100 %
Dynamic Mode	Standard

Physio - PACE

Resp. Control	Off
Concatenations	1

Inline - Subtraction

Subtract	Off
Measurements	1
StdDev	Off
Save Original Images	On

Inline - Cardiac

Magn. Preparation	None

Inline - Cardiac

Save Original Images	On
TE	11.00 ms
TR	700.0 ms

Inline - MIP

MIP Sag	Off
MIP Cor	Off
MIP Tra	Off
MIP Time	Off
Radial MIP	Off
Save Original Images	On
MPR Sag	Off
MPR Cor	Off
MPR Tra	Off

Inline - Composing

Inline Composing	Off

Sequence - Part 1

Sequence Name	spcR
Dimension	3D
Excitation	Non-sel.
RF Pulse Type	Normal
Gradient Mode	Fast
Flow Compensation	None
Reordering	Radial
Bandwidth	630 Hz/Px
Echo Spacing	3.82 ms
Turbo Factor	38
Echo Train Duration	157 ms

Sequence - Part 2

Introduction	On

Sequence - Assistant

SAR Assistant	Off
Allowed Delay	30 s
