# Supplementary material for: The Austrian MS database and the Austrian MS cohort: A national effort towards data harmonization and prospective data collection
Source: Wien Klin Wochenschr. 2026 Jan 12;138(13-14):394–402. doi: 10.1007/s00508-025-02689-2 (PMC13375655; doi:10.1007/s00508-025-02689-2)
Supplement: Supplementary file 3 — Overview of laboratory parameters collected in the Austrian Multiple Sclerosis Cohort (AMSC) [file 508_2025_2689_MOESM3_ESM.docx]

| **Monitoring for MS within AMSC/AMSD** | | | | | | | | |
| --- | --- | --- | --- | --- | --- | --- | --- | --- |
|  |  |  |  |  |  |  |  |  |
| **DMT start** | **General** | **GLAT IFNb** | **DMF TERI** | **S1PM, CLA**  **NTZ, CD20, ATZ** | | | | |
|  |  |  |  |  | | | | |
|  | CBC, differential CBC | 0 | Hep. B/C | Hep. B/C  HIV  VZV  Quantiferon  Lues  Ig quantitativ  JCV | | | | |
|  | Chemistry panel |  | HIV |  |  |  |  |  |
|  | Coagulation tests |  | VZV |  |  |  |  |  |
|  | Leucocyte typing |  | Quantiferon |  |  |  |  |  |
|  | (+ CD 19/20) |  |  |  |  |  |  |  |
|  | Urine status |  |  |  |  |  |  |  |
|  | Vaccination status |  |  |  |  |  |  |  |
|  | Biobank |  |  |  |  |  |  |  |
|  | Height and weight |  |  |  |  |  |  |  |
|  | Blood pressure (BP) |  |  |  |  |  |  |  |
| **DMT monitoring** | **General** | **GLAT IFNb** | **DMF TERI** | **S1PM** | **CLA** | **NTZ** | **CD20** | **ATZ** |
|  |  |  |  |  |  |  |  |  |
|  | CBC, differential CBC | 0 | BP | BP | 0 | JCV | Hep. B/C | Urine status |
|  | Chemistry panel |  |  |  |  |  | HIV |  |
|  | Coagulation tests |  |  |  |  |  | Ig quantitativ |  |
|  | Leucocyte typing |  |  |  |  |  |  |  |
|  | (+ CD 19/20) |  |  |  |  |  |  |  |
|  | Biobank |  |  |  |  |  |  | Biennially |
|  | Weight |  |  |  |  |  |  | Thyroid |
|  |  |  |  |  |  |  |  |  |
| Vaccine status | Hep. B quantitative |  |  |  |  |  |  |  |
|  | FSME Titer (IgG) |  |  |  |  |  |  |  |
|  | Measles IgG |  |  |  |  |  |  |  |
|  | Mumps IgG |  |  |  |  |  |  |  |
|  | Rubella IgG |  |  |  |  |  |  |  |
|  |  |  |  |  |  |  |  |  |

ATZ: alemtuzumab. CD20: B cell deleting antibodies (rituximab, ocrelizumab, ofatumumab, ublituximab). CLA: cladribine. DMF: dimethylfumarate. GLAT: glatirameracetate. IFNb: interferon beta. NTZ: natalizumab. S1PM: sphingosin 1 receptor modulator (fingolimod, siponimod, ozanimod, ponesimod). TERI: teriflunomide.

| **Monitoring for NMOSD/MOGAD within AMSC/AMSD** | | | | |
| --- | --- | --- | --- | --- |
|  |  | | | |
| **DMT start** | **General** | **Rituximab / Uplizna** | **Ultomiris / Soliris** | **Tocilizumab / Enspryng** |
|  | CBC, differential CBC | IL-6 | IL-6 | IL-6 |
|  | Chemistry panel | CH50 | CH50 | CH50 |
|  | Coagulation tests |  |  | Cholesterine |
|  | Leucocyte typing (+ CD 19/20) |  |  | LDL |
|  | Hep. B/C; HIV, VZV |  |  | Trigylceride |
|  | Vaccination status |  |  | Fibrinogene |
|  | Lues |  |  |  |
|  | Quantiferon |  |  |  |
|  | Ig quantitative |  |  |  |
|  | JCV |  |  |  |
|  | Urine status |  |  |  |
|  | AQP4/MOG |  |  |  |
|  | Biobank |  |  |  |
|  | Height and weight |  |  |  |
| **DMT monitoring** | **General** | **Rituximab/ Uplizna** | **Ultomiris / Soliris** | **Tocilizumab / Enspryng** |
|  | CBC, differential CBC | Hep. B/C | IL-6 | IL-6 |
|  | Chemistry panel | HIV | CH50 | CH50 |
|  | Coagulation tests | IL-6 |  | Cholesterine |
|  | Leucocyte typing (+ CD 19/20) | CH50 |  | LDL |
|  | Ig quantitative |  |  | Trigylceride |
|  |  |  |  | Fibrinogene |
|  | AQP4/MOG **(once per year)** |  |  |  |
|  | Biobank |  |  |  |
|  | Weight |  |  |  |
|  |  |  |  |  |
| Vaccine status | Hep. B quantitative |  |  |  |
|  | FSME Titer (IgG) |  |  |  |
|  | Measles IgG |  |  |  |
|  | Mumps IgG |  |  |  |
|  | Rubella IgG |  |  |  |
